# Design, structural, spectral, DFT and analytical studies of novel nano-palladium schiff base complex

**DOI:** 10.1038/s41598-022-21406-x

**Published:** 2022-10-19

**Authors:** Magda A. Akl, Nora A. El-Mahdy, El-Sayed R. H. El-Gharkawy

**Affiliations:** Chemistry Department, Faculty of Science, Mansoura University, Mansoura, 35516 Egypt

**Keywords:** Nanoscale materials, Theory and computation, Nanoscience and technology, Environmental sciences, Environmental chemistry

## Abstract

A novel nano-palladium (II) Schiff base complex (C1) is synthesized by the reaction between palladium chloride and the Schiff base N, N’-1, 2-phenylene) bis (3 -aminobenzamide (A1). The prepared compounds were characterized by elemental analysis, Ultraviolet–Visible spectroscopy (UV–Vis), Fourier transform infrared spectroscopy (FTIR), Scanning electron microscopy (SEM), Transmission electron microscopy (TEM) and Thermogravimetric Analysis (TGA). A combined solvent sublation-ICP OES methodology has been studied for the preconcentration, separation and determination of trace palladium (II) in media of diverse origin using the Schiff base ligand (A1). The different experimental variables that affect the sublation efficiency (*S*, %) were thoroughly investigated *viz.*: pH of sample solution; amounts of A1, Pd (II) and TBAB; type and amounts of surfactants, types of organic solvent, temperature and stirring time. The method involves the determination of trace palladium (II) after selective separation by solvent sublation, thus eliminating the effect of foreign ions and increasing the sensitivity. Also, palladium is determined directly in the organic phase, which decreases the determination time and its loss during determination. At optimum conditions, the linear range of Pd (II) was 10.0–100.0 ngmL^−1^. The coefficient of determination, the limit of detection (LOD) and limit of quantification (LOQ) were 0.9943, 21.29 ngL^−1^ and 64.5 ngL^−1^, respectively. This sublation method was applied to real samples and recoveries of more than 95% were obtained in the spiked samples with a preconcentration factor of 100. The mechanism of solvent sublatation of the TBA.[Pd^II^-(A1)_2_] ion pairs is discussed. The computational studying was estimated to approve the geometry of the isolated solid compounds.

## Introduction

Palladium, as a precious metal, plays a very important role in modern industry due to its attractive physical and chemical properties such as excellent corrosion resistance, stable thermoelectricity and high catalytic activity. Palladium has been widely used in in electronics industry, in the production of dental and medicinal devices, in hydrogenation, dehydrogenation and organic synthesis and in automotive catalytic converters. It is reported currently that more than 50% of the world’s production of palladium are being consumed in production of auto-catalysts each year^1^ Due to the extended use of palladium in modern industries, the emission of this metal into the environment has increased considerably^2^ Under appropriate conditions, such as pH and redox potential, it is assumed that palladium undergoes methylation reactions in the aquatic environment and could be concentrated along the food chain, resulting in ecological and human health risks^3,4^ Consequently, establishing simple, highly sensitive and selective method for the determination of trace amounts of palladium in water samples is of great importance. However, it is often not possible to make direct determinations of palladium by employing the known analytical techniques due to its low or even extremely low concentration as well as matrix effects^1^ Pre-concentration and separation coupled with highly sensitive technique is one of the best ways to solve these problems. There are many approaches for preconcentration and separation, such as liquid–liquid extraction ^5,6^ solid phase extraction^7−9^, ion exchange^10,11^ and HPLC^12^ and CPE^13^

A chelate extraction system is widely used for the pre-concentration of trace elements prior to atomic absorption spectrometric (AAS) determination. Hydrophobic chelating extractants have gained great interest for the separation of metal ions^14^. A flotation technique is also useful to separate inorganic ions from large volumes of sample solutions. Precipitate and ion flotation techniques have been developed to determine many kinds of elements with a high concentration ratio^15–20^. Recently, a solvent sublation technique^21,22^ has been developed and used as a combined method of flotation and solvent extraction^23^ with the advantages of both techniques.

Schiff base ligands provide a great platform in coordination chemistry for the development of numerous ligand systems with controllable binding to metal ions^24–26^. Ligands in this class could potentially stabilize metals in different oxidation states and induce stability in homogeneous and heterogeneous catalysts, which is particularly useful when they are viewed in catalytic activity perspectives^27^. Metal complexes derived from Schiff bases have found applications in different research areas, including and not limited to, molecular magnetism, catalysis and medical sciences^28^. Despite abundant reports regarding Schiff bases as ligands in the coordination chemistry of transition metals and their diverse applications, no research has been reported regarding palladium complexes supported with Schiff base ligands for solvent sublation of Pd (II). The present study is based on the fact that platinum group elements easily form their complexes with thio-organic compounds or long-chained alkyl amines^29^.

Bearing in mind the mentioned importance of palladium complexes and as a continuation of our recently published work^30^, the synthesis and characterization of nano-palladium (II) complex (C1) with the Schiff base N, N’-1, 2-phenylene) bis (3-aminobenzamide ligand (A1) are described in this paper.

A literature survey showed that no attempt has been found for using N, N’-1, 2-phenylene) bis (3-aminobenzamide Schiff base ligand (A1), TBAB, oleic acid surfactant (HOL) and MIBK in solvent sublation-ICPOES determination of palladium (II). In the present work, a number of experimental variables have been evaluated, e.g. pH, concentration of metal and ligand, temperature, etc. In addition to measurements of palladium (II) in synthetic sample, other samples such as river, seawater samples, were also analyzed. Moreover, density functional theory (DFT) studies were carried out to rationalize the experimental work and support the obtained results.

## Experimental

### Reagents and solutions

All the solvents and chemicals used were of analytical reagent grade and were used without purification. All glass wares were dipped into bidistilled water and dried in an oven at 80 °C. Palladium (II) chloride hydrated PdCl_2_.2H_2_O and % tetrabutylammonium bromide (TBAB) (99.5% purity) were obtained from Sigma Aldrich. Stock solution of 1 × 10^−4^ M Pd (II) was prepared by dissolving 0.00106 g PdCl_2_.2H_2_O in 100 ml of bidistilled water by adding 1.0 ml of concentrated HCl. Stock solution of 1 × 10^−3 M^ of the Schiff base ligand (A1) chelating agent was prepared by dissolving 0.04385 g Schiff base ligand (A1) in 100 ml of ethanol. A stock solution of 6.36 × 10^−2^ M oleic acid (HOL) was prepared from the food-grade with sp. gr. 0.895 by dispersing 20 ml of HOL in one liter of kerosene. A stock solution of 0.1% tetrabutylammonium bromide (TBAB) was prepared by dissolving the solvent in bidistilled water.

### Instruments

- For the solvent sublation technique, two types of flotation cells were used^31^. Type 1 was a cylindrical graduated glass tube of 16 mm inner diameter and 290 mm length with a stopper at the top and a stopcock at the bottom. This cell was used for adjusting the factors affecting the efficiency of the sublatation process. The second one was a cylindrical tube of 6.0 cm inner diameter and 45 cm length with a stopcock at the prime and was used for the separation of the studied analytes from the quite large volumes.
- Elemental analyses (C, H, N, M) were performed using a Costech ECS- 4010- Analyzer.
- The Fourier transform-infrared (FT-IR) spectra were recorded with a JASCO FT/IR-460 spectrophotometer with the use of KBr tablets in the range from 400 to 4000 cm^−1^ at room temperature.
- Ultra violet visible (UV–Vis) spectra were gained for the prepared ligand (A1) and the complex (C1) by using a PerkinElmer 550 spectrophotometer in 1 cm quartz cell in ethanol over a range of 200–900 nm.
- Traces of Pd (II) were detected by inductivity coupled plasma–optic emission spectroscopy (ICPOES) through a Varian spectrometer Model Varian Vista Pro, CCD Simultaneous. The optimum parameters are shown in (Table S1).

### Preparations

#### Synthesis of the Schiff base ligand (N, N’-1, 2-phenylene) bis (3-aminobenzamide) (A1)

**Figure 1:**
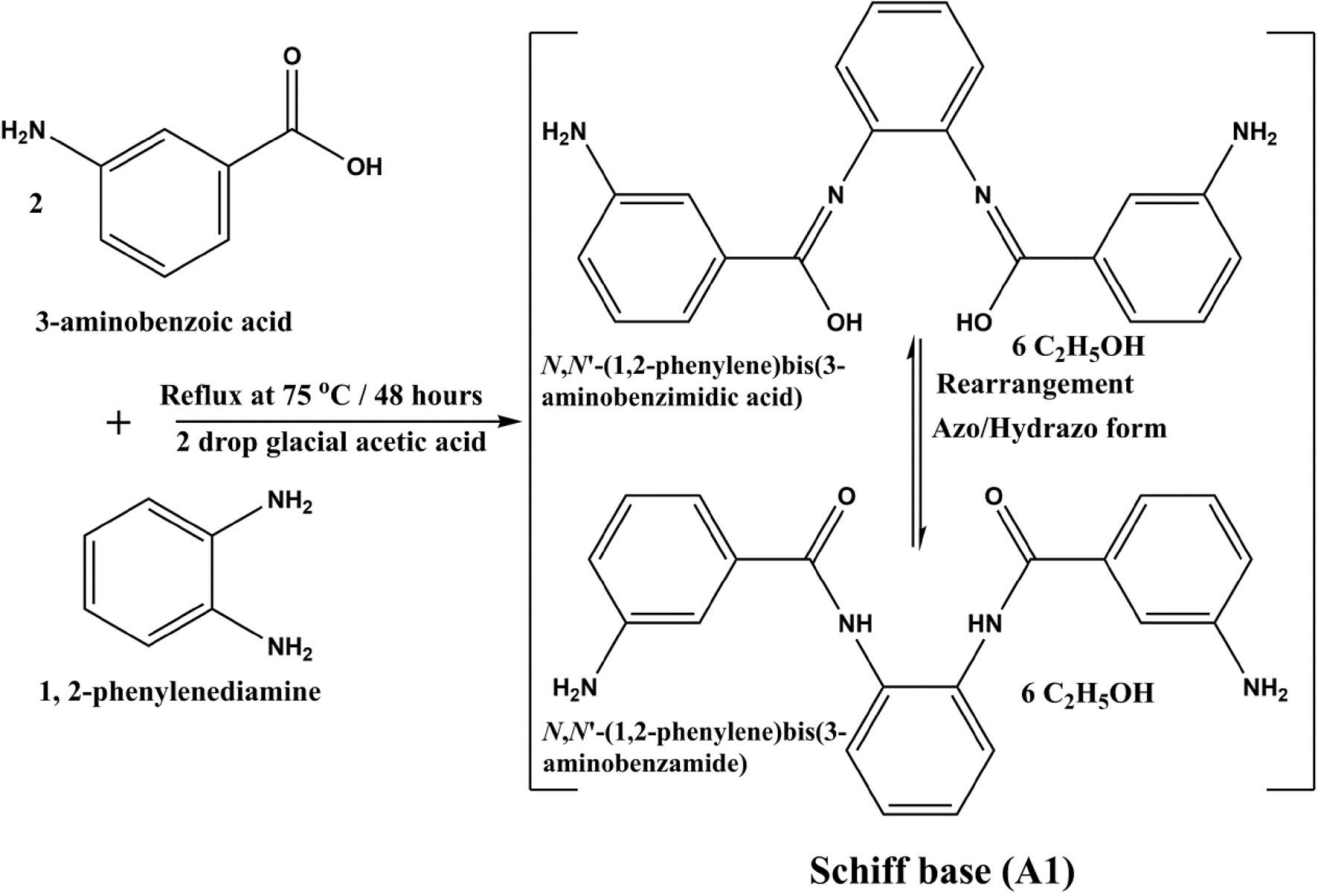
Synthesis of the Schiff base ligand (A1).

The Schiff base (A1) is prepared according to our recently published work^30^. The Schiff base (A1) was produced from refluxing a 3-aminobenzoic acid (3-ABA) (2.0 mmol, 0.2743 g) dissolved in 10 ml ethanol with 5 ml of 1, 2-phenylenediamine (Phen) (1.0 mmol, 0.1081 g) dissolved in ethanol in the presence of 2 drops of glacial acetic acid (1 M) for 2 days at 75 °C according to reaction synthesis^32,33^, Fig. 1. During the reaction, the color of the prepared solution was changed from the pale yellow to the formed reddish-brown precipitate. This formed precipitate was filtered off, washed several times with water/ ethanol, and afterward dried under a vacuum.

#### Synthesis of the nano-palladium (II) Schiff base Complex (C1)

Nano-palladium Schiff base complex (C1) was synthesized via refluxing PdCl_2_.2H_2_O (1.0 mmol, 0.2134 g) dissolved in 10 ml distilled water (DW) with ligand (A1) for 2 days at 75 °C. Afterwards the system was refluxed. During the reaction, the color of the solution was changed from reddish-brown to a dark red precipitate. The precipitate was filtered off, washed several times with water/ ethanol, and finally dried under a vacuum.

### Molecular modeling

Geometry optimizations and other DFT calculations were performed on the Schiff base (A1) ligand and its palladium complex (C1) using B3LYP level of theory, Becke’s three parameter (B3) non local exchanges with the correlation functional of Lee, Yang, and Parr (LYP)^34^. We used the B3LYP level to study electronic compounds because the predicted geometries are very reliably and provide good estimations for HOMO–LUMO gaps, in a good agreement with experimental values^30,35–41^. All the calculations were carried out by using 6–31 g (d) basis set for O, N, C and H atoms and lanl2dz basis set was used for Pd atom. All computations were carried out by using Gaussian09 suite of program^42^. Gauss View 5.0 package was used to obtain various graphic views of molecular shapes of distinctive molecular.

### Solvent sublation of palladium (II)

In an Erlenmeyer flask, 2 mL of 1.88 × 10^−5^ mol L^−1^ (A1) solution was added to an aliquot containing 0.94 × 10^−6^ molL^−1^ of Pd (II); the pH was adjusted by 0.5 mol L^−1^ HCl and or 0.5 mol L^−1^NaOH and the solution was mixed completely. A red color of C1 developed instantaneously. Two ml of 4 × 10^−4^ molL^−1^ of TBAB solution were added and the mixture was shaken well for 2 min to allow complete development of the TBA.[Pd^II^-(A1)_2_] ion pairs. All contents were quantitatively transferred into a flotation cell (type 1) and the volume was adjusted to 10 mL. Then, 2 mL of 2 × 10^−4^ mol L^−1^ HOL was added. The flotation cell was shaken upside down for 2 min. Vigorous shaking of the flotation cell in the presence of the surfactant (HOL) created bubbles in the solution which enhanced the floatability of the TBA.[Pd^II^-(A1)_2_] ion pairs. 5 mL of MIBK were added to the solution surface and the flotation cell was shaken upside down by hand. The red TBA.[Pd^II^-(A1)_2_] ion pairs were quantitatively extracted into the organic layer on the solution surface. The aqueous phase was run off through the bottom of the cell.

After complete sublatation (10 min) the scum layer was evaporated to half volume by heating in a water bath. It was then made up to 10 mL in a volumetric flask using a solution containing 20:80, 1.0 mol L^−1^ HNO_3_/10% v/v methanol and analyzed by ICP OES to measure the concentration of Pd(II) at λ_max_ 340.4 nm with axial viewing.

The solvent sublation efficiency (S %) of Pd (II) was determined from the following relationship:

1 $$S\% \; = \;\left( {C_{o} /C_{i} } \right)\; \times \;100$$

Here, C_o_ and C_i_ denote the concentration of Pd (II) in the organic and in the initial aqueous layers, respectively.

Alternatively, Pd (II) was determined directly by ICP OES in the aqueous mother liquor. The separation efficiency (S %) of the analyte was calculated from its concentration in the mother liquor according to the relation

$$S \, = \, \left[ {\left( {c_{i} - c_{f} } \right)/c_{i} } \right] \, x \, 100 \, \%$$

where *c*_*i*_ and *c*_*f*_ denote the initial and final concentrations of the analyte, respectively.

## Results and discussion

### Ligand, metal-complex and ion pairs

Both A1 and C1 are stable colored compounds, slightly soluble in DMF and soluble in DMSO. The most important obtained physical and microanalytical data for the prepared compounds are summarized in Table 1. Melting point is ~ 174.1 °C for A1 and > 300 °C for C1; the yields of A1 and C1 are 96.5 and 82.1%, respectively.

**Table 1:** Analytical and some important physical measurements for A1, C1 and C2.

C1	A1	Assignments	
Refluxing	Refluxing	Preparation method	
Dark red	Reddish-brown	Color	
Fine crystal	Powder	Aspect	
> 300	174.1	Melting point (°C)	
48 h	48 h	Reaction time	
86.4	96.5	Yield (%)	
C_24_H_28_N_4_O_4_Pd	C_24_H_30_N_4_O_4_	Chemical formula	
542.93	438.53	Molecular weight (g/mol)	
(53.09)/53.19	(65.73)/65.94	C%	**Elemental analysis (Calc.)/Foun**d
(5.20)/5.31	(6.90)/6.73	H%	
(10.32)/10.22	(12.78)/12.89	N%	
3451–3275	3635–3278	ν(OH)–ν(NH) azo hydrazine form	**Characteristic infrared frequencies (cm**^**−1**^**)**
1636	1730	ν(C = O)	
1454	1470	ν(C = N)	
1345	1440	ν(C = C)	
1253	1385	ν(C–N)	
671	–	ν(Pd–O)	
436	–	ν(Pd–N)	
236, 268, 274, 510	226, 296, 440	UV‐λ_max_ (nm)	

C1	C2	Assignments	
3000–3700		ν(OH)–ν(NH)–	**Characteristic infrared frequencies (cm**^**−1**^**)**
		ν(NH_2_)	
1750–1440		ν(C = O)	
		ν(C = N)	
1354		ν(C–N)	
671		ν(Pd–O)	
436	2900	ν(Pd–N)	
		ν(CH) of TBAB	

A1 is dissociated to A1^−^ and H^+^ and the unshared electron pairs of –O– and = N– in A1 takes part in a coordination bond with metal ions. Anionic metal complex is formed in the presence of excess A1; and the anionic complex forms ion pairs with TBA^+^ ion by the addition of tetra-*n*-butyl ammonium bromide solution^43^.

### Characterization

#### Elemental analysis

The elemental analyses of the Schiff base A1, the Pd(II)-A1 complex (C1) and the [TBA-Pd(II)-A1] ion pairs (C2), are presented in Table 1. The obtained results were in agreement with that calculated for the suggested formula. Anal. Calc. (%): for A1; C_24_H_30_N_4_O_4_; (438.53 g/mol); C, 65.73; H, 6.90; N, 12.78; found C, 65.94; H, 6.73; N, 12.89. Whereas, Anal. Calc. (%): for C1; C_24_H_28_N_4_O_4_Pd; (542.93 g/mol); C, 53.09; H, 5.20; N, 10.; found C, 53.19; H, 5.31; N, 10.22.

#### FT-IR Spectra

**Figure 2:**
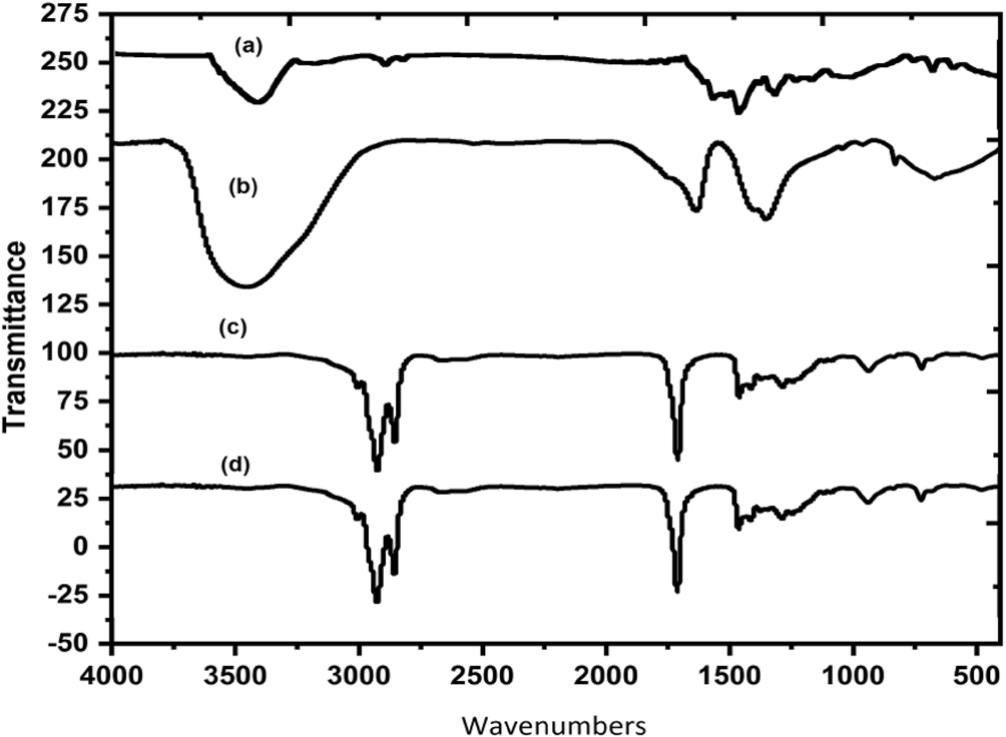
The FTIR spectra of (**a**) the ligand A1, (**b**) C1 complex in aqueous solution, (**c**) TBA.[Pd^II^-(A1)] (C2) ion pair in aqueous layer and (**d**) TBA.[Pd^II^-(A1)](C2) ion associate in organic layer.

The FTIR spectra of the ligand A1, the complex C1 in aqueous solution, TBA.[Pd (A1)] (C2) ion associate in organic layer are presented in Fig. 2a,b,c,d. The most significant frequencies of A1, C1 and C2 are presented in Table 1. As it has already been mentioned earlier, the IR of A1(Fig. 2a) exhibits an absorption broad band in the range between 3635–3278 cm^−1^ which can be recognized to the υ(OH) of the alcohol, υ(OH), υ(NH) of keto-enol form and υ(NH_2_) ^44,45^. The band at 2935 cm^−1^ is assigned to υ(CH)-sp3. The bands between 1730–1440 cm^−1^ can be attributed to υ(C = O), υ(C = N) and ν(C = C) and the broad bands due to the formation of the two forms of keto and enol structures. The band that appeared at 1385 cm^−1^ is due to ν(C–N) groups. The bands between 1330 and 736 cm^1^ are due to aromatic CH^46^. The IR spectrum of the C1 complex, Fig. 2b, proves the complexation of the ligand A1 with the metal ion. In contrast to the spectrum of the free ligand A1, the IR of the complex C1 shows a significant shift of υ(OH),ν(NH), υ(C = N) and υ(C–N) vibrations. While, the υ(C = O) and υ(C = C) vibrations stay at more or less at the same place, which designates that (C = C) does not contribute in coordination with the metal ion. So, A1 acts as a neutral bidentate coordination site via υ (OH), (C–N), (C = O), and azomethine nitrogen (C = N) atoms. This observation is additionally supported by new bands that were found at 671 and 436 cm^−1^ that can be assigned to υ (Pd–O) and υ(Pd–N), respectively^47^.

**Figure 3:**
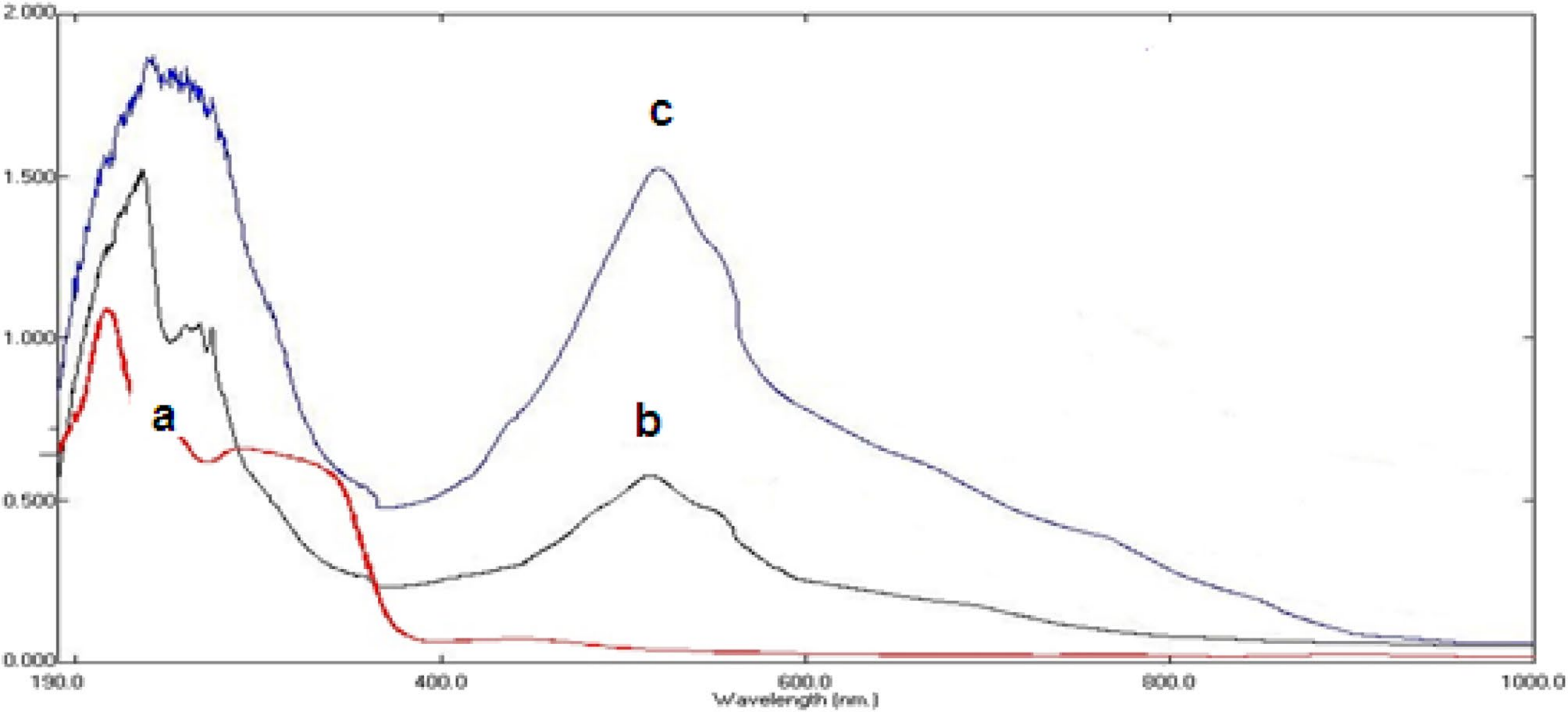
The electronic absorption spectra of (a) Schiff base ligand (A1), (b) Pd complex ( C1) in aqueous solution and (c) TBA.[Pd (A1)] ion associate in organic layer.

The IR spectrum of the C2 ion pair in aqueous solution, Fig. 2c, is different from the IR spectra of A1 and C1, Fig. 3a,b, reflecting that there is chemical contribution between TBAB and the C1 complex as –NH_2_ stretching band disappeared and new –NH stretching band appeared. This means that protonation occurred by the release of hydrogen atom of (–NH_2_) carrying positive charge with Br^−^ atom of TBAB carrying negative charge, so electrostatic bonding has been formed.

Finally it was noticed that the IR spectrum of the TBA.[ Pd^II^-(A1)] (C2) ion pair isolated in the aqueous solution, (Fig. 2c) and in the organic layer, (Fig. 2d) are identical denoting that the process of sublation could be physical in nature.

#### UV–vis spectra

The electronic absorption spectra of A1 and C1were showed in Fig. 3. From Fig. 3, it was recognized that the A1 (Fig. 3a) exposes three absorption bands at 226, 296, and 440 nm, which were agreed to intra ligand charge transfer (n–π* and π–π*). While, the C1 exposes four absorption bands at 236, 268, 274, and 510 nm, which were agreed to the ligand–metal charge transfers transitions (LMCT) and owing to the intra-ligand charge transfers (n–π* and π–π*)^48–52^.

The absorption spectra of the C1 in the aqueous solution and in the organic layer are completely different from that of A1. It is observed that: 1) the λ_max_ of C1 exhibits a red shift (70 nm) from that of A1 and 2) the absorbance of C1-HOL-MIBK system exhibits nearly 4-folds that of C1 in aqueous solution. This proves that the species are highly concentrated in the presence of HOL and MIBK.

#### Scanning electron microscopy (SEM) and Transmission electron microscopy (TEM)

**Figure 4:**
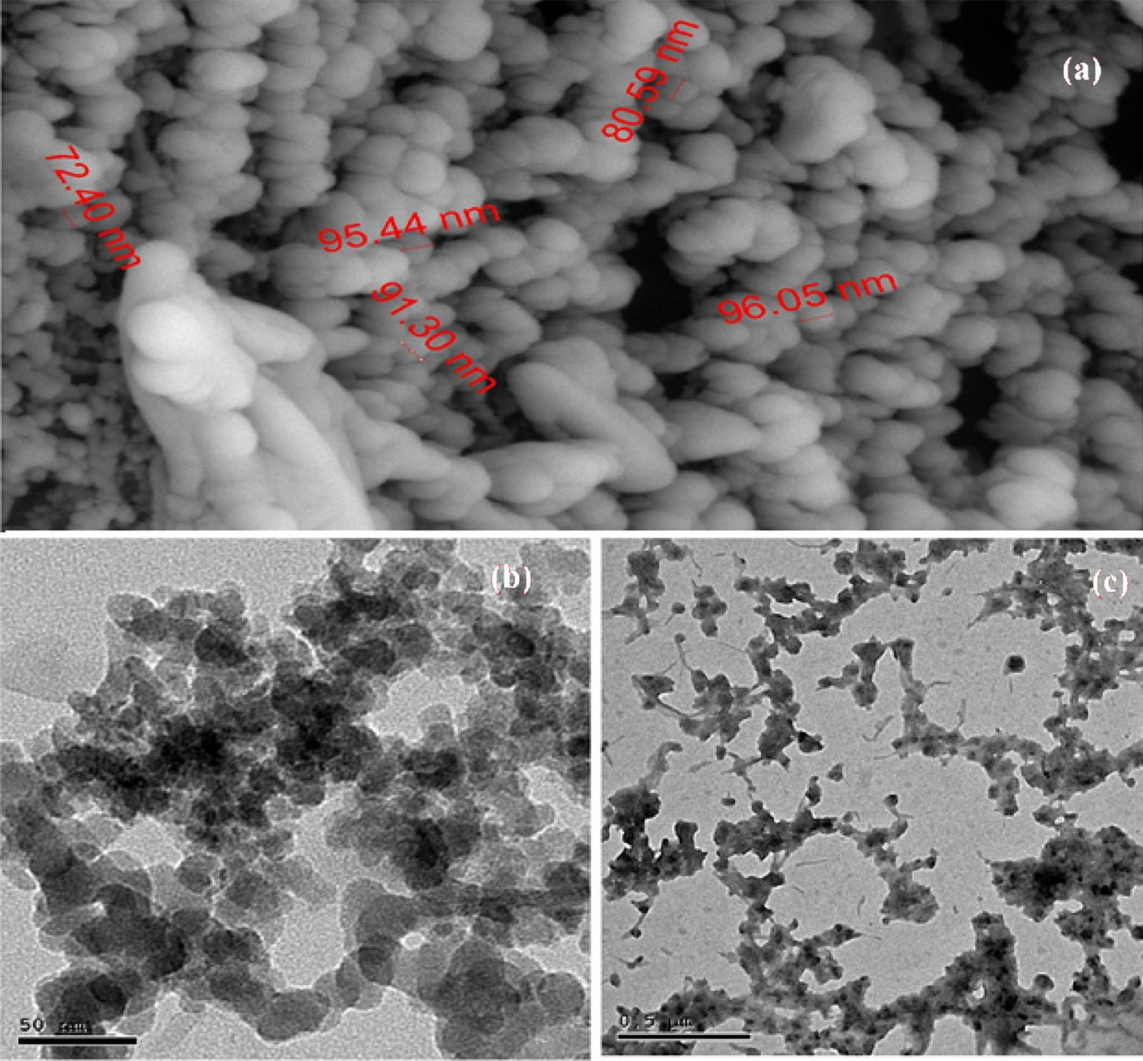
**a** SEM of C1; **b** & **c** TEM of C1.

Scanning electron microscopy (SEM) and Transmission electron microscopy (TEM) were employed to characterize the micromorphology and the size and shape of nanoparticles. As shown in the SEM image (Fig. 4a), the nano-palladium (II) complex (C1) complex nanoparticles were present as uniform particles with spherical and ovoid morphology. From the TEM micrographs (Fig. 4b,c), the average particle size of C1 nanoparticles is estimated between 72.0 to 97.0 nm.

### Geometry optimization with DFT calculations

**Figure 5:**
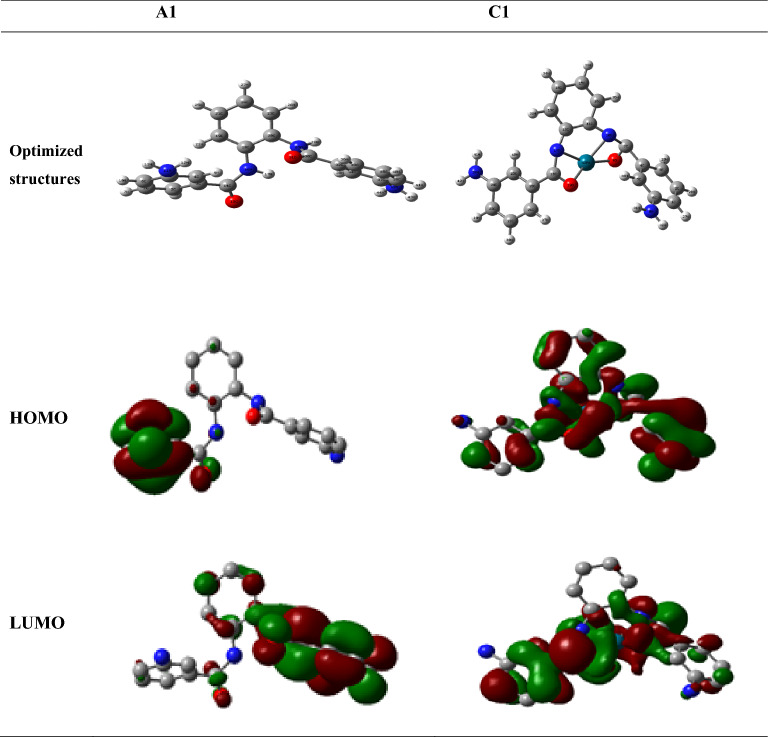
Optimized structures, HOMO and LUMO based on DFT calculations by using B3LYP method with 6–31 g (**d**) basis sets.

The optimized geometry of the Schiff base (A1) ligand and its palladium complex (C1) are shown in Fig. 5. Highest Occupied Molecular Orbitals (HOMO) and Lowest Unoccupied Molecular Orbitals (LUMO) are very considerable elements of theoretical molecular design^53^. The HOMO is electron donor and LUMO the electron acceptor sites and the molecular hardness and softness of a compound can be predicted from the HOMO–LUMO gap^54^.

The Global Reactivity Parameters^39–41^ such as ionization potential (I_P_), electron affinity (E_A_), hardness ($\eta )$, softness ($\sigma$) and electronegativity ($\chi$)] can be determined from the HOMO and LUMO orbital energies through Koopman’s theorem ^55^. The ionization potential (I_P_) and electron affinity (E_A_) are defined as the negative of HOMO and LUMO energies, respectively (I_P_ =  − E_HOMO_ and E_A_ =  − E_LUMO_). The chemical hardness ($\eta )$ is a measure of resistance of an atom against the charge transfer ^56^. It can be computed using the I_P_ and E_A_ energies:

$$\eta = \frac{{I_{P} - E_{A} }}{2}$$

The softness ($\sigma$), which is the reciprocal of hardness ($\sigma = \frac{1}{\eta }$), is a property of its capability to accept the electron.

Thus, a structure becomes harder (softer) when its energy gap (ΔE_(LUMO–HOMO)_) is getting wider (narrower). The electronegativity ($\chi$) in terms of IP and EA are given by:

$$\chi = \frac{{I_{P } + E_{A} }}{2}$$

The higher reactivity of the Pd-complex (C1) over the Schiff base ligand (A1) is explained in the light of energy gap, ΔE_(LUMO–HOMO)_, which measures the reactivity; as the energy gap decreases the reactivity increases and the amount of electronic charge transferring from (A1) to the central metal ion increases. Table 2 illustrates that ΔE _(LUMO–HOMO)_ for Pd-complex (C1) was found to be more reactive than of the Schiff base ligand (A1). Hard molecules ($\eta )$ have a large energy gap, and soft molecules ($\sigma$) have a small energy gap^57,58^. A soft molecule is more reactive than a hard molecule because a soft molecule has a lower ΔE _(LUMO–HOMO)_^59^. From Table 2, C1 is softer than A1 and this confirms that C1 is more reactive than L1. The $\chi$ is a measure of power of atom(s) to attract the electrons^60^. A high value of electronegativity (*χ*) for C1 suggests strong ability to attract electrons from the A1, which leads to greater interaction to form the complex C1. Finally, the dipole moment (μ) is a factor that can also provide information about interaction between A1 molecule and Pd. The value (μ) of Pd–complex (C1) is higher than (μ) of the Schiff base ligand (A1); this suggests the stronger interactions between A1 and Pd to form the complex. It was observed that there is charge density transferred from Schiff base atoms to metal ion and there is electrons back-donation from the metal ion to the donating atoms on the Schiff base after complexation. The (E_HOMO_), (E_LUMO_), energy gap ΔE_(LUMO–HOMO)_, ionization potential (I_P_), electron affinity (E_A_), hardness ($\eta$, softness ($\sigma$), electronegativity ($\chi$) and dipole moment (μ) of the Schiff base (A1) and the Pd-complex (C1) are listed in Table 2.

**Table 2:** Global Reactivity Parameters determined using DFT calculations for Schiff base (A1) and Pd-complex (C1).

Parameter	Schiff base (A1)	Pd-complex (C1)
E_HOMO_ [eV]	− 0.202	− 0.200
E_LUMO_ [eV]	− 0.037	− 0.162
$\Delta E_{(LUMO - HOMO)}$ [eV]	0.166	0.038
I_p_ [eV]	0.202	0.200
E_A_ [eV]	0.037	0.162
(η) [eV]	0.083	0.019
(σ) [eV]^−1^	12.083	52.356
$\chi$ [eV]	0.120	0.181
µ [Debye]	3.422	6.694

### Solvent sublation and ICP OES determination of palladium (II)

Solvent sublation depends on the solubility of the metal-complex in an organic solvent. But the solvent should be immiscible with an aqueous phase, non-volatile and lighter than the aqueous solution. It must be kept at the surface of the solution in a stable state. The sublation efficiencies were compared with several water-immiscible and light solvents such as methylisobutyl ketone (MIBK), diisopropyl ketone (DIPK), cyclohexane, benzene and o-xylene, having 0.77–0.89 g/mL densities. MIBK showed good sublation efficiencies for similar levels and was used in subsequent studies.

#### Method development

Before analysis of the standard and real samples by ICP OES, convenient conditions of the instrument should be obtained. Thus, different parameters influencing the intensity of signals in ICP OES such as radio frequency (rf), generator power, nebulizer pressure, viewing height of plasma, auxiliary gas flow rates and peristaltic pump rate were optimized. Then, to guarantee that maximum extraction efficiency was obtained, the parameters affecting solvent sublation efficiency including metal, surfactant and chelating agent concentration, shaking time and temperature, pH of solution and sample volume were optimized. The optimized conditions of ICP OES are given in Table S1.

#### Digital photographs

**Figure 6:**
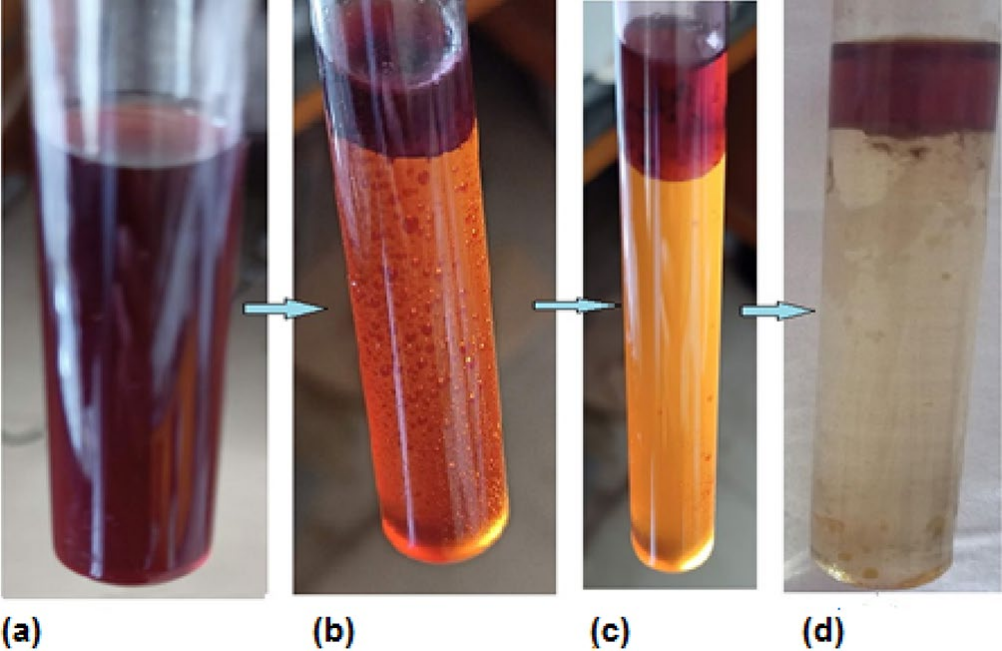
Digital photographs of the sublation process. **a**) TBA.[Pd^II^-(A1)] ion pair in aqueous solution; **b**) & **c**) TBA.[Pd^II^-(A1)] ion pair after addition of HOL; **d**) TBA.[Pd^II^-(A1)] ion pair sublated to the MINK organic layer.

The digital photographs of the TBA.[Pd^II^-(A1)] ion pairs in the aqueous solution and in the organic layer are shown in Fig. 6. As it can be clearly seen, the ion pair has a clear homogenous red color in the aqueous solution (Fig. 6a). After the successful sublation of the ion pair, the red colored TBA.[Pd^II^-(A1)] ion pair is concentrated in the organic layer on the top of solution surface while the rest of the solution becomes colorless as it can be noticed in Fig. 6d.

#### Effect of experimental variables

##### Effect of pH

**Figure 7:**
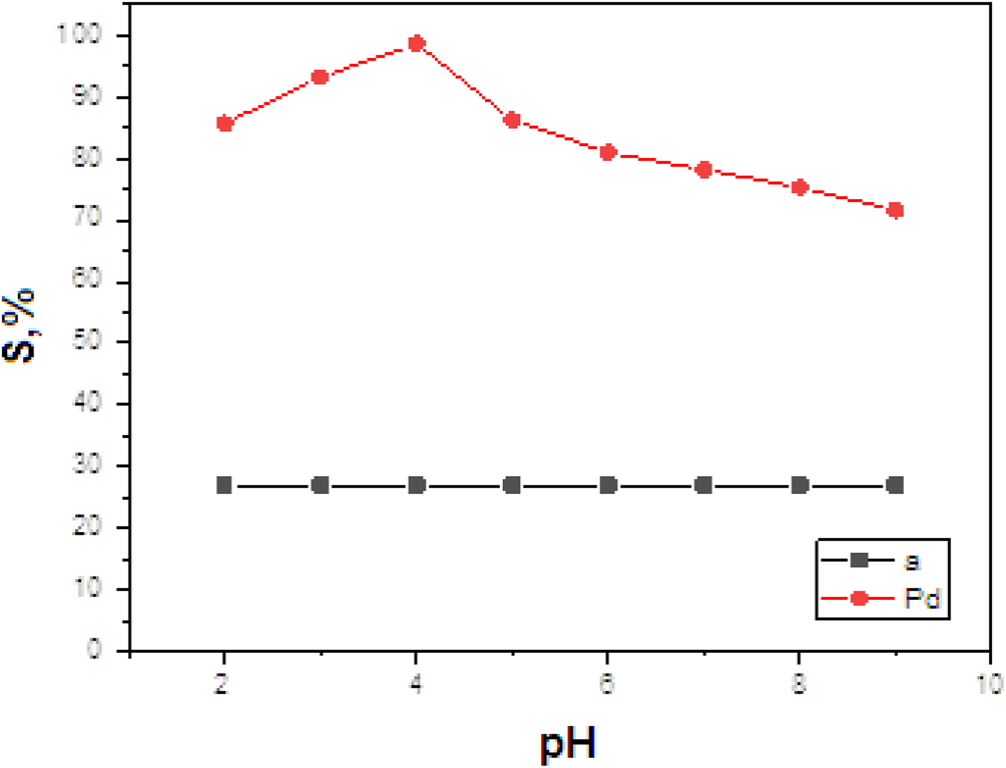
Effect of pH on sublation efficiency of Pd (II); (**a**) in the absence of A1; (**b**) in the presence of 2.0 × 10^−4^ mol L^−1^ A1 by using 5 × 10^−4^ mol L^−1^ TBAB; 2 × 10^−3^ mol L^−1^ HOL; MIBK (5 ml) at room temperature.

The formation of C1 complex was influenced by solution pH and Pd (II) ions can form hydroxide precipitates in a strongly basic solution. The completeness of complex formation was investigated by changing the pH from 2 to 7 (Fig. 7). The sublation of the Pd (II) was very low in the acidic range of pH 2–3 because the analyte could not be extracted into MIBK. This is due to the difficult formation of complexes because A1 is nearly non ionized in such an acidic solution. Hydroxide precipitates also were not formed in this range. A series of experiments was carried out to study the effect of pH on the sublation efficiency (S%) of Pd (II) (2 × 10^−6^ mol L^−1^) with 5 ml of 1 × 10^−3^ mol L^−1^ TBAB, 2 × 10^−3^ mol L^−1^ HOL and 5 ml MIBK in the absence and in the presence of 2 × 10^− ^mol L^−1^ of A1. The data in Fig. 7, curve a, prove that in the absence of the ligand A1, the maximum separation efficiency doesn't exceed 30% at all pH values tested. Such separation percent is not satisfactory from the analytical point of view. On the other hand, Fig. 7, curve b, shows that complete separation (≈100%) of Pd (II) was obtained at pH 4.0 in the presence of 2 × 10^−4^ mol L^−1^ of A1. Further experiments were carried out at pH 4.0.

##### Effect of concentration of A1 and Pd (II)

**Figure 8:**
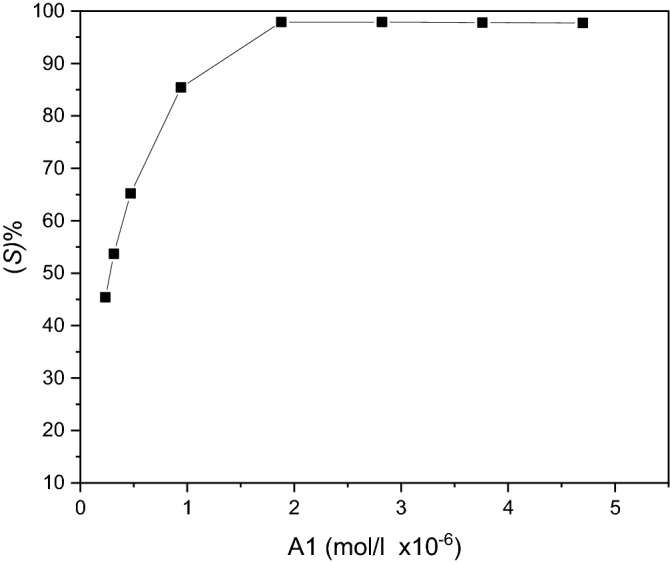
Influence of A1 concentration on the sublation efficiency of 1.0 × 10^−6^ mol L^−1^ Pd (II) at pH 4.0 using 5 × 10^−4^ mol L^−1^TBAB; 2 × 10^−3^ mol L^−1^ HOL; MIBK (5 ml).

The effect of ligand concentration on the sublation efficiency (S% ) of a 1.0 × 10^−6^ mol L^−1^ of Pd(II) was investigated at pH 4.0 using 5 ml of 1 × 10^−3^ mol L^−1^ TBAB, 2 mL of 2 × 10^−3^ mol L^−1^ HOL, 5 ml MIBK and various amounts of A1. The sublation efficiency (S%) increases with increasing ligand concentration untill it reaches its maximum at 2.0 × 10^−6^ mol L^−1^ of A1; at this concentration the molar ratio is 1:2 Pd (II):A1. The results given in Fig. 8 show that excess ligand amount had no drastic effect on the sublation process.

To confirm the previous data, another series of experiments was carried out by fixing the ligand concentration and changing the metal concentration from 1.0 to 5.0 × 10^−6^ mol L^−1^). The results obtained showed that complete separation occurs at the same previous ratio of 1:2 Pd (II): A1. Above such a ratio, increasing Pd (II) concentration results in decrease of the separation efficiency. This reflects that insufficient ligand is present for complete complexation and indirect separation.

##### Effect of TBAB concentration

As described earlier, Pd (II) ions form anionic complex with an excess of A1 and this anionic complex forms ion pairs with the TBAB. The amount of the TBAB affects the stability of the TBA.[Pd^II^-(A1)] ion pair. Under other optimum conditions, the efficiency of solvent sublation of 1.0 × 10^−6^ mol L^−1^ Pd (II) was investigated by changing the TBAB concentration in the presence of 2.0 × 10^−5^ mol L^−1^ of A1, 2 × 10^−3^ mol L^−1^ HOL and 5 ml MIBK at pH 4.0. The results in Fig. 1S, show that the maximum sublation efficiency (S %) of TBA. [Pd^II^-(A1)] was attained when the concentration of TBAB was ≥ 2 × 10^–4^ mol L^−1^. Thus, a 5 ml of 4 × 10^−4^ mol L^−1^ of TBAB was chosen in the present study.

##### Type and amount of surfactant

In general, a surfactant is added to float some materials in an aqueous solution by making them hydrophobic. Hydrophobic materials can be more effectively seeded from aqueous solutions than hydrophilic materials. The material can be a precipitate, complex ions or ion associate (ion pairs) species. TBA. [Pd^II^-(A1)] was the ion pair in the present sublation methodology.

In the present study, the efficiency of sublation by the type of surfactant was evaluated using anionic (HOL), non-ionic TX-80 and the cationic (CTAB) surfactants. The TBA.[Pd^II^-(A1)] ion associate was not sublated at all upon using the cationic or the nonionic surfactants. On the other hand, the sublation efficiency (S %) reaches its maximum on using the HOL anionic surfactant. Such phenomenon can be interpreted to mean that excess TBA^+^ around Pd^II^-(A1)^−^ complex creates the ion pairs of positive charge. Therefore, the addition of the anionic surfactant improves the efficiency of sublation.

The concentration of HOL is an important parameter; up to a limit the separation percentage increases as the concentration of the surfactant increases. The effect of HOL concentration on the sublation efficiency of TBA.[Pd^II^-(A1)] ion pairs were investigated. The results obtained indicated that the sublation efficiency of TBA.[Pd^II^-(A1)] ion pair reaches its maximum (100%) over a wide range of HOL concentrations (1 × 10^**−**3^ to 1 × 10^**−**4^ mol L^**−**1^) until its critical micelle concentration (CMC) is reached. At a higher HOL concentration, there will be a concentration, at which the surfactant molecules gather together to form a microball, called ***micelle*.** Micelles compete for the ion pairs and since they stay in solution, they reduce the effectiveness of separation. The concentration of the surfactant also changes the bubble size with the size getting smaller as the surfactant increases. This makes creamier foam. A suitable concentration (2X10^**−**3^ mol L^**−**1^) of HOL was selected throughout this work.

##### Effect of temperature and shaking time

To study the role of temperature on the Pd (II) separation, Pd (II), surfactant and A1 solutions were either heated or cooled to the same temperature. A1, TBAB and HOL are quickly poured into the Pd (II) solution at zero time. The solution was introduced into flotation cell Jacketed with 1 cm thick fiberglass insulation. The sublation steps were preceded as previously mentioned at pH≈4. The results obtained, Fig. 2S, indicated that the sublation of TBA.[Pd^II^-(A1)] is not affected by raising the temperature up to 40 °C above which the sublation efficiency starts to decrease. The decrease in the sublation efficiency upon increasing the temperature may be attributed to the instability of the ion pairs TBA.[Pd^II^-(A1)] as well as the weak binding between the HOL surfactant and the ion associate species. A finding, that helps to explain the mechanism of sublation of the TBA.[Pd^II^-(A1)] ion associate species by the HOL surfactant and MIBK. The whole work has been carried out at room temperature (25 ± 1 °C).

A proper shaking is required for complete complexation of A1 with palladium ions, which forms hydrophobic complex after around 2.0 min and remains constant until 48.0 h, as shown in Fig. S3. For the following work, a shaking time of 60.0 s followed by a rest period of 3.0 min was used.

##### Influence of sample volume

A series of experiments were conducted to sublate a fixed concentration of the analyte (1.0 × 10^−6^ mol L^−1^ of palladium) from different aqueous volumes (50–1500 ml) using suitable large flotation cells under the recommended conditions. It is clear from the data in Fig. 4S that, 10^−6^ mol L^−1^ of the analyte can be quantitatively separated from different aqueous volumes up to 1 L using 10 mL of the organic layer. The enrichment factor (preconcentration factor)^61^, defined as the ratio of the volumes before and after solvent sublation, was 100.

##### Influence of ionic strength

Table 2S summarizes the effect of ionic strength on the separation efficiency of Pd (II) under the recommended conditions. Sodium, magnesium and calcium as chloride and sulfate were added during the sublation separation of the analyte at the recommended conditions. It was found that these salts, even up to 0.5 mol L^−1^ concentration have no effect on the solvent sublation efficiency of the analyte.

##### Influence of different ions

In the view of the high selectivity provided by ICP-OES, the interferences studied were related to the pre-concentration step. The effect of foreign metal ions on the solvent sublation of Pd (II) with A1 has been studied in detail. The obtained results reveal that in spite of the high tendency of A1 to form complexes with different transition metal ions, fortunately, most of these complexes are not sublated with the Pd (II) complex at pH 3.5–4.0. The experimental data showed that although some foreign ions have little interfering effects (∼3%), Table 3, all of these interferences were completely controlled by adding excess A1 (2 × 10^−4^ mol L^−1^). Hence, one can predict that the interfering effects may be due to complex formation which is accompanied by a decrease in the ligand concentration. Consequently, masking of the interfering effects by adding excess A1 offers a highly selective procedure for the separation and determination of microamounts of Pd (II) in various complex materials, like environmental and biological samples.

**Table 3:** Tolerance limits for the determination of 1.0 × 10^−6^ mol L^−1^ Pd (II).

Foreign ions	Concentration (mg L^−1^)	S (%)
Pb^+2^	5.0	99.1
Ni^+2^	5.0	97.3
Zn^+2^	5.0	95.5
Mn^+2^	10.0	98.3
Hg^+2^	5.0	96.6
Ba^+3^	10.0	98.3
Cr^+3^	10.0	99.1
Cu^+2^	3.0	98.3
Fe^+3^	3.0	97.6
Co^+2^	3.0	98.1
Bi^+2^	3.0	98.3
Al^+3^	3.0	98.9
Na^+^	230.0	98.7
Cl^−^	177.5	99.1
CH_3_COO^−^	295	98.23
NO_2_^−^	230	99.45
NO_3_^−^	310	99.62

##### Analytical characteristics

Under the optimum conditions, the calibration graph was linear in the range 10.0–1000.0 ng mL^-1^. The calibration equation is y = 2.6696x (μg mL^−1^) + 0.0415, with a correlation coefficient of 0.9943. The limit of detection^62^, (defined as LOD = 3 SB/m, where SB and m are standard deviation of the blank and the slope of the calibration graph, respectively), was 21.29 ngL^−1^. The limit of quantification (LOQ) calculated as 3 LOD was 64.5 ngL^−1^. The relative standard deviation was 0.023% (c = 5.0 μg mL^−1^, n = 3). The analytical characteristics of this technique are shown in details in Table 4.

**Table 4:** The analytical characteristics of the method.

Parameter	Pd (II)
Linear range (ng/mL)	10.0–1000
SD	0.01723
RSD% (n = 3)	0.023
LOD (ng/L)	21.29
LOQ (ng/L)	64.5
Pre-concentration factor	100
Correlation coefficient	0.9943
Regression equation*	y = 2.6696x + 0.0415

### Analytical applications

The optimized experimental conditions were applied to real samples to evaluate the efficiency of the combined solvent sublation-ICP-OES method for the pre-concentration and determination of trace Pd (II) ions. The calibration curves were prepared with the standard solutions. The standard solutions (1.0 L) were treated under the experimental conditions optimized above.

#### Determination of Pd (II) in synthetic sample and palladium catalyst waste water

The proposed method was applied to determination of trace amounts of palladium in synthetic samples and the palladium catalyst waste water with satisfactory results, Table 5.

**Table 5:** Determination of Pd (II) in synthetic sample and palladium catalyst waste water by ICP OES after solvent sublation using 2 × 10^−4^ mol l^−1^ A1, and 2 × 10^−4^ mol l^−1^ HOL, and 5 ml MIBK at pH 4.0. (*n* = 5).

Sample	Determined (μg mL^−1^)	Added (μg mL^−1^)	Found (μg mL^−1^)	RSD (%)	Recovery (%)
**Palladium catalyst waste water**	35.25	4.00	4.02	1.24	100.5
		8.00	7.92	0.49	99.0
		12.00	11.86	0.82	98.8
**Synthetic Sample** [500.0 μg mL^−1^ of K^+^, Mg^2+^, Co^2+^, Mg^2+^, Ca^2+^, Ni^2+^ and 20 μg mL^−1^ of Pt (IV), Ir (IV), Au (III)]	15.00	4.00	3.89	1.03	97.3
		8.00	7.89	1.21	98.6
		12.00	11.84	1.08	98.7

#### Determination of Pd (II) in natural water samples

In order to investigate the applicability to a natural-water samples, the recoveries of known amounts of Pd (II) added to bidistilled, river and sea water samples were examined by such a procedure. To 20 ml aliquots of clear uncontaminated, filtered water samples 1, 3 and 5 μg of Pd (II) were added and the pH was adjusted by HCl to pH 4.0. The as previously mentioned solvent sublation –ICP OES methodology was applied. The recoveries obtained, Table 6, were in the range of 98.00–99.42%. These results indicate that this analytical method could be successfully applied to the determination of trace Pd (II) in real water samples.

**Table 6:** Determination of Pd(II) in natural water samples by ICP OES after solvent sublation using 2 × 10^−4^ mol l^−1^ A1, 5 × 10^−4^ mol L^−1^ TBAB, 2 × 10^−4^ mol l^−1^ HOL and 5 ml MIBK at pH 4.0 at room temperature. (*n* = 5).

Types of water (location)	Spiked (μg ml^−1^)	Measured	Recovered (μg ml^−1^)	Recovery (%)	RSD (%)
Distilled water (Our lab)	0.00	0.00	0.00	0.00	0.00
	1.00	0.991	0.991	99.1	1.9
	3.00	2.952	2.952	98.4	1.2
	5.00	4.935	4.935	98.7	1.5
Tap water (Our lab)	0.00	0.00	0.00	0.00	0.00
	1.00	0.981	0.981	98.1	1.3
	3.00	2.97	2.97	99.0	1.22
	5.00	4.915	4.915	98.3	1.21
Nile water (Mansoura city)	0.00	0.00	0.00	0.00	0.00
	1.00	0.98	0.98	98.00	1.54
	3.00	2.946	2.946	98.2	1.52
	5.00	4.90	4.90	98.00	1.61
Seawater (Alexandria city)	0.00	0.00	0.00	0.00	0.00
	1.00	0.982	0.982	98.2	1.72
	3.00	2.973	2.973	99.1	0.85
	5.00	4.971	4.971	99.42	1.41

### Mechanism of solvent sublation of [TBA^+^[Pd^II^-(A1)_2_^−^] ion-associate complex

According to Sebba^63^, the separation mechanism in the solvent sublation technique is very simple. As the gas bubbles pass through the liquid mass they collect the colligend-collector species, which is then transferred to the organic phase on the upper surface of the liquid mass. In studies concerning separation via sublation, the role of surfactant is very important. The nature of the interaction between oleic acid surfactant and the formed TBA^+^Pd^II^-(A1)_2_^**−**^ ion-pair must be studied to approach the actual mechanism of sublation. An ion pair is a pair of oppositely charged ions held together by Coulomb attraction without formation of a covalent bond. The proposed mechanism may proceed through: i) a physical interaction through Van der Waal; ii) by forming a hydrogen bond between the hydrophilic part of the surfactant and the active sites in the ligand complex or iii) by an interaction between oleic acid and the ion pair, formed in solution through a coordinate bond, forming a self-floatable [TBA^+.^[Pd^II^-(A1)^**−**^-HOL) species. In all cases, the hydrophobic part of the surfactant attaches to air bubbles and floats separating the analyte-containing species.

As it has been mentioned earlier in this study, A1 forms anionic complex with Pd (II) and the addition of sufficient amount of TBAB turned this anionic complex into a positively charged ion pair that was easily made hydrophobic by the addition of HOL, floated by vigorous shaking of the flotation cell and extracted into the MIBK.

In the present investigation, the mechanism of sublation is proposed to be physical in nature. This suggestion may be attributed to the following findings:

1. The sublated species in the organic layer has the same colour (red) as that obtained in the aqueous solution (especially in high reactants concentrations).
2. The sublation efficiency was greatly affected by temperature. Raising the temperature above 40 °C results in a marked depression in the sublation efficiency of the TBA.[Pd^II^-(A1)] ion associate complex. This behavior can be attributed to the weak binding between the ion pair and oleic acid reflecting that there is no coordinate or hydrogen contribution between HOL and the TBA.[Pd^II^-(A1)] ion associate. Also, the physical force between HOL and TBA.[Pd^II^-(A1)], can easily be destroyed by heat.
3. Finally it was noticed that the IR spectra of the TBA.[Pd^II^-(A1)] ion associate isolated in the aqueous solution, (Fig. 2c) and organic layer, (Fig. 2d), are identical denoting that the process of sublation could be physical in nature.

In conclusion, all of the mentioned studies suggest the first proposal mechanism.

### Comparison with published reports

A comparison between the analytical performances of the present approach and other previous reports in the kinds of literature for the determination of palladium is presented in Table 7. The analytical figures of merits of our work are comparable or better than previously reported reports.

**Table 7:** Comparative data from some recent studies on preconcentration-separation of palladium ions.

Methods	Instrumentation	EF	LOD (µg/L)	RSD%	Reference
CPM	ICP OES	20.2,8.6	0.3	5	^64^
SPE	FAAS	572	0.08	3	^65^
SPE	FIA-FAAS	–	17	1.2	^66^
CPM	ICP MS	40,35	0.06	4.1, 5.4	^67^
SPE	FASS	75	–	1.7	^68^
DLLME	ETAAS	48.7	4.8 ng/L	3.1	^69^
SPE	FAAS	400	100	4.1	^70^
DLIME	GFAAS	388	5	1.4	^71^
DLIME	FAAS	113	0.4	2, 2.4	^72^
Solvent sublation	ICP OES	100	21.29 ng L^−1^	0.023	This work

## Conclusion

In this paper, nanopalladium(II) complex of the 1-N, N’-1, 2-phenylene)bis(3-aminobenzamide) Schiff base ligand (A1) was synthesized and characterized by elemental analysis, spectroscopy techniques and physical measurements*.* The results showed that the A1acted as a neutral bidentate ligand. Trace Pd(II) in several water samples of adjusted pH 4.0 were determined by a solvent sublation using an ion pair of Pd^II^(A1)^−^ anion and tetrabutylammonium ion for the utilization of a synergistic effect. The hydrophobic ion pairs created by the addition of oleic acid were floated and extracted into MIBK by vigorous shaking of the flotation cell. This procedure was applied to the analysis of several real water samples and recoveries of more than 95% were obtained in spiked samples of given amounts of analytes. The RSD of less than 3% was obtained for standard spiked samples. Such results show that this is a fairly accurate and reproducible method and can be applied to similar samples. The results show that the solvent sublation pretreatment is a sensitive, rapid, simple and safe method for the separation/preconcentration of palladium. Also, DFT calculations were done to predict the host-gust interaction between the Schiff base and Pd (II) cations.

## Supplementary Information

Supplementary Information.

## Data Availability

All data generated or analysed during this study are included in this published article.
