# Supplementary material for: Design, structural, spectral, DFT and analytical studies of novel nano-palladium schiff base complex
Source: Sci Rep. 2022 Oct 19;12:17451. doi: 10.1038/s41598-022-21406-x (PMC9582206; doi:10.1038/s41598-022-21406-x)
Supplement: Supplementary file 1 — Supplementary Information. [file 41598_2022_21406_MOESM1_ESM.doc]

**Supplementary materials**

**Design, Structural, Spectral, DFT and Analytical Studies of Novel Nano-palladium Schiff base complex**

**Magda A Akl*^1^, Nora A El-Mahdy^1^ and El-Sayed R H El-gharkawy^1^**

^1^Chemistry Department, Faculty of science, Mansoura University, Mansoura, Egypt

*Corresponding author: Chemistry Department, Faculty of science, Mansoura University, Mansoura, Egypt; e-mail: magdaakl@yahoo.com

**
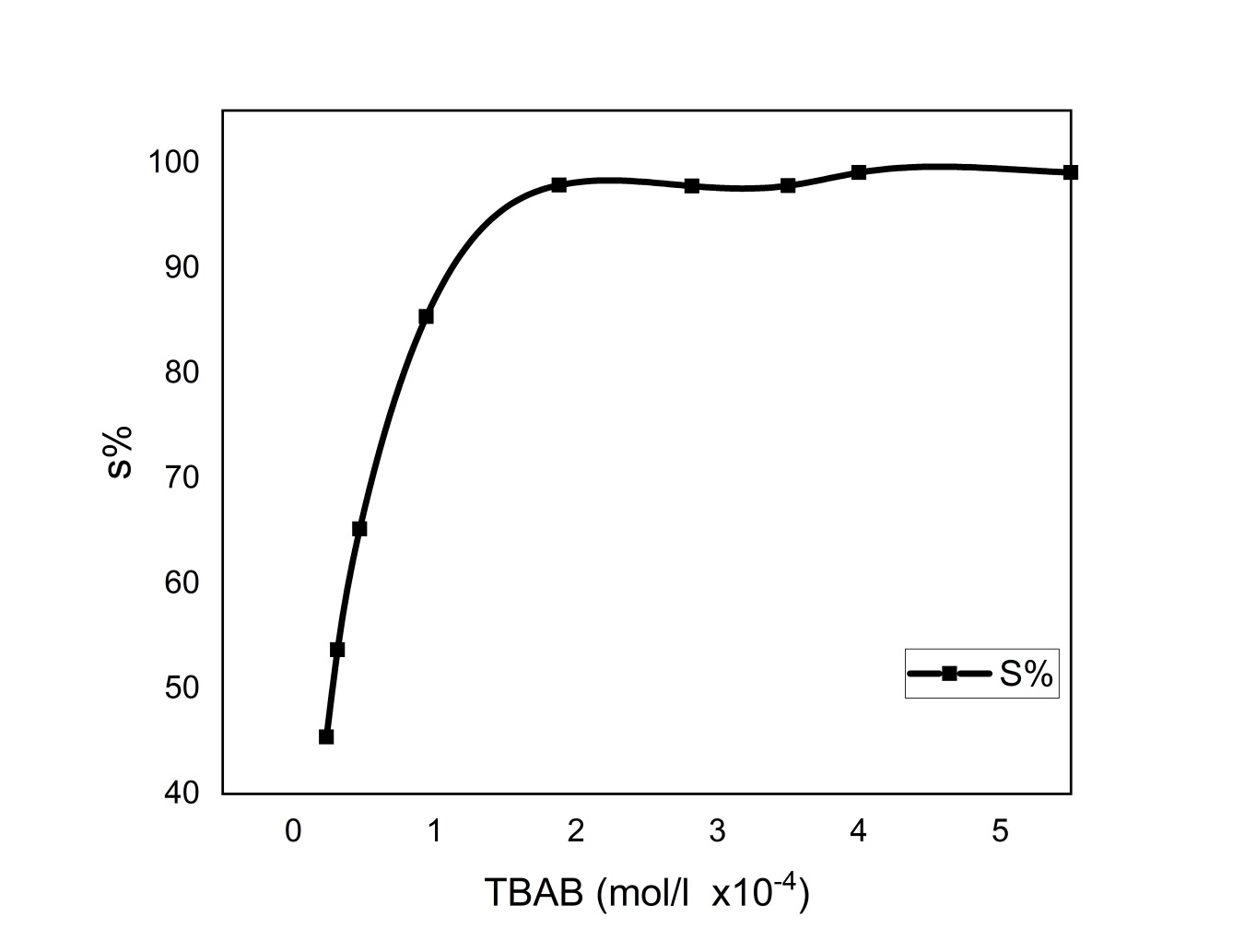
**

**Fig.1S** Influence of TBAB concentration on the sublation efficiency (*S*, %) of 1x10^-6^ mol L^-1^ Pd (II) at pH 4.0, using 2x10^-5^ mol L^-1^ A1, 2×10^-3^ mol L^-1^ HOL; MIBK (5ml).


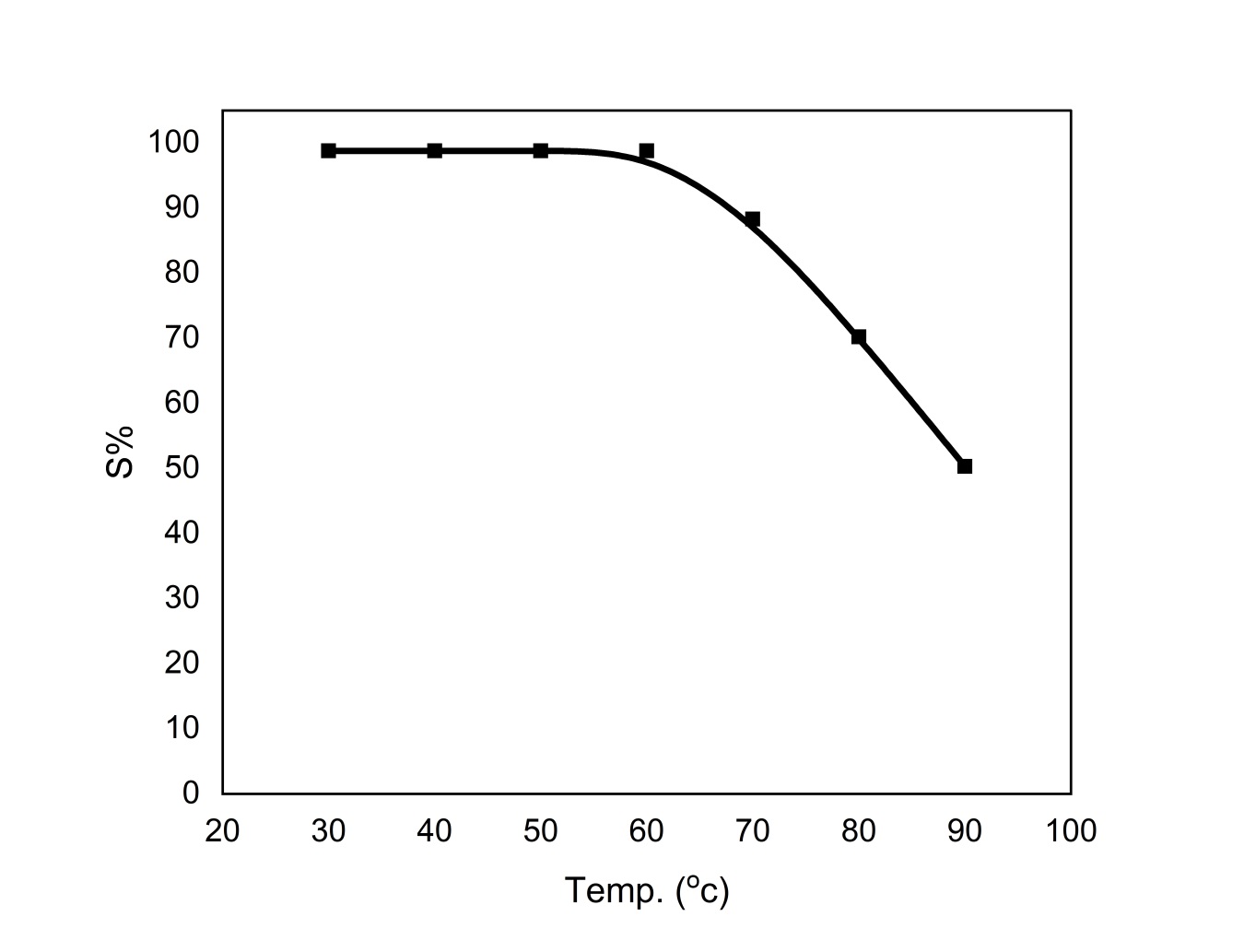


**Figure 2S** Influence of temperature on sublation efficiency of 1.0x10^-6^ mol L^-1^ analyte at pH 4.0 in the presence of 2x10^-5^ mol L^-1^ A1 using 5×10^-4^ mol L^-1^ TBAB; 2×10^-3^ mol L^-1^ HOL; MIBK(5ml).

**Figure 3S** Effect of time on sublation efficiency of 1.0x10^-6^ mol L^-1^ Pd (II) at pH 4.0 in the presence of 2.0 x10^-5^ mol L^-1^ A1 using 5×10^-4^ mol L^-1^ TBAB; 2×10^-3^ mol L^-1^ HOL; MIBK (5ml).

**Fig. 4S** Effect of sample volume on Flotation efficiency of 1.0x10^-6^ mol L^-1^ analyte in the presence of 2.0x10^-5^ mol L^-1^ A1,5×10^-4^ mol L^-1^ TBAB; 2×10^-3^ mol L^-1^ HOL, MIBK (5ml)

**Table 1S**

**The optimized conditions for ICP OES determination of Pd(II)** ions

| RF generator power (kW) | 1.2 | Pd wavelength (nm) | 340.458 |
| --- | --- | --- | --- |
| Frequency of RF generator (MHz) | 40.68 | Viewing height (mm) | 9 |
| Plasma gas flow rate (l min^−1^) | 12 | Pump rate (rpm) | 15 |
| Auxiliary gas flow rate (l min^−1^) | 0.75 | Viewing | axial |
| Nebulizer pressure (kPa) | 160 | λ _max_ | 340.4 nm |

Table 2S

Influence of ionic strength on the sublation efficiency of Pd(II). Conditions: Pd(II): 2x10^-6^ mol L^-1^, A1: 2x10^-4^ mol L^-1^, TBAB. 1x10^-3^ mol L^-1^; HOL 2x10^-3^ mol L^-1^, MIBK (5 mL) and pH 4.0 at ~25^o^C).

| Ionic Strength, mol L^-1^ |  | Salt |  | S, % |
| --- | --- | --- | --- | --- |
|  |  |
| 0.01 |  | NaCl |  | 100.05 |
|  |  | Na_2_SO_4_ |  | 99.90 |
|  |  | CaCl_2_ |  | 99.50 |
|  |  | MgCl_2_ |  | 100.0 |
| 0.10 |  | NaCl |  | 99.30 |
|  |  | Na_2_SO_4_ |  | 99.80 |
|  |  | CaCl_2_ |  | 100.00 |
|  |  | MgCl_2_ |  | 99.70 |
| 050 |  | NaCl |  | 98.9 |
|  |  | Na_2_SO_4_ |  | 99.10 |
|  |  | CaCl_2_ |  | 98.5 |
|  |  | MgCl_2_ |  | 98.60 |
